# Non-invasive prenatal testing for the detection of trisomy 13, 18, and 21 and sex chromosome aneuploidies in 68,763 cases

**DOI:** 10.3389/fgene.2022.864076

**Published:** 2022-09-15

**Authors:** Yanchun Zhang, Hongyan Xu, Wen Zhang, Kaibo Liu

**Affiliations:** ^1^ Department of Perinatal Health, Beijing Obstetrics and Gynecology Hospital, Capital Medical University, Beijing, China; ^2^ Department of Perinatal Health, Beijing Maternal and Child Health Care Hospital, Beijing, China

**Keywords:** noninvasive prenatal testing, karyotype analysis, sensitivity, specificity, positive predictive value

## Abstract

**Objectives:** Non-invasive prenatal testing (NIPT) has been widely used in recent years. According to clinical experience from all hospitals providing prenatal screening services in Beijing, we explored the feasibility of using NIPT for the analysis of common foetal aneuploidies among pregnancies.

**Methods:** In total, 68,763 maternal blood samples were collected from January 2020 to December 2020 at the Beijing prenatal diagnosis agency. Cases with positive screening results by NIPT detection were validated using prenatal diagnosis.

**Results:** In total, 920 cases had a high-risk NIPT result, and 755 cases were shown to be truly positive by a chromosome karyotyping analysis; the prenatal diagnosis rate was 82.07% (755/920). Of the920 cases, there were 164 cases of T21, 70 cases of T18, 38 cases of T13, 360 cases of SCAs and 288 cases of other chromosomal abnormalities. The positive rates of T21, T18, T13, and SCAs were 0.24% (164/68,763), 0.10% (70/68,763), 0.06% (38/68,763) and 0.52% (360/68,763), respectively. The sensitivity and specificity were 98.17% and 99.92% for T21, 96.15% and 99.93% for T18, and 100% and 99.95% for T13, respectively. The PPVs of T21,T18,T13 and SCAs were65.24% (107/164), 35.71% (25/70), 18.42% (7/38) and 31.39% (113/360), respectively. For all indications, there were more higher T21/18/13 in the high-risk group than in the low-risk group (comprising only cases of voluntary request), with a positive rate of 0.46% vs. 0.27% (*p* < 0.001), sensitivity of 99.16% vs. 91.30% (*p* = 0.02) and PPV of 56.73%vs.32.81% (*p* = 0.001), but there was no significant difference in specificity between the groups (*p* = 0.71). The detection indication with the highest PPV (100%) by NIPT was ultrasound structural abnormalities and ultrasound soft marker abnormalities for T21 and ultrasound structural abnormalities and NT thickening for T18 and T13. The PPVs of different clinical indications of T21 (*p* = 0.002), T13 (*p* = 0.04) and SACs (*p* = 0.02) were statistically significant.

**Conclusion:** The high specificity, efficiency and safety (non-invasiveness) of NIPT can effectively improve the detection rate of common chromosomal aneuploidy, thereby reducing the occurrence of birth defects. We should encourage pregnant women with NIPT-high-risk results to undergo a prenatal diagnosis to determine whether the foetus has chromosomal abnormalities. More importantly, the screening efficiency of NIPT in the low-risk group was significantly lower than that in the high-risk group. Therefore, the use of NIPT in low-risk groups should be fully promoted, and socioeconomic benefits should be considered.

## Introduction

Non-invasive prenatal testing (NIPT) using cell-free foetal DNA in maternal plasma has been successfully employed for aneuploidy screening in clinical settings for 10 years (Hartwig et al., 2017). Several studies have assessed the accuracy of this method based on actual clinical experience. Indeed, many studies have demonstrated that NIPT can reduce the incidence of unnecessary invasive procedures (Zhang et al., 2015). NIPT also has higher sensitivities and specificities than traditional biochemical and sonographic screening. Previous studies have reported detection and false-positive rates of 99.2% and 0.09% for trisomy 21 (T21), 96.3% and 0.13% for trisomy 18 (T18), and 91.0% and 0.13% for trisomy13 (T3) (Gil et al., 2017). Additionally, other studies have reported detection rates above 98% for trisomy 21 and above 93% for trisomy 18 (Health Quality Ontario, 2019). In the study by Taylor-Phillips et al. (2016), the sensitivities for trisomy 21, trisomy 18 and trisomy13 were 99.3%, 97.4% and, 97.4%, respectively. Overall, NIPT is an accurate screening test that offers the opportunity to improve the detection of aneuploidies while reducing the use of invasive diagnostic procedures. Nevertheless, previous studies have reported that with a cell-free foetal DNA (cffDNA) fraction of less than 4% (American College of Obstetricians and Gynecologists Committee on Genetics, 2012), the foetal-placental DNA is indistinct, and a false-negative NIPT is likely to occur due to placental mosaicism (Hartwig et al., 2017). In addition, one study concluded that 45% of chromosomal abnormities could not be detected by cffDNA testing but could be detected by invasive diagnostic procedures with cytogenetic analysis (Shani et al., 2016).

Here, we explore the use of NIPT from the perspective of evidence-based clinical medicine. We collected all NIPT and prenatal diagnosis results from all prenatal diagnosis institutions in the entire city to evaluate the screening efficacy of NIPT in Beijing. Our research was approved by our institutional ethics committee.

## Materials and methods

### Patients

A total of 68,763 women from all delivery institutions in Beijing accepted NIPT; 755 underwent cytogenetic prenatal diagnosis at eight prenatal diagnosis centres from January 2020 to December 2020. All of the pregnant women who participated in the study received prenatal genetic consultation and then voluntarily signed informed consent forms, which were issued by the General Office of the National Health and Family Planning Commission. We divided the patients into a high-risk group and a low-risk group according to the screening indications of NIPT. High-risk groups included the indications of advanced maternal age, high risk of serological screening, critical risk of serological screening, NT thickening, ultrasound soft marker abnormality, ultrasound structural abnormality, twin/IVF–ET pregnancy, missed time for serological screening, contraindications for interventional surgery and others according to Document No. 45 of National Health and Family Planning Commission (National Health and Family Planning Commission, 2016) and Taylor-Phillips S, et al. (Taylor-Phillips et al., 2016).

The low-risk group included only voluntary screening indications.

### Prenatal screening and diagnosis

#### Non-invasive prenatal testing

**FIGURE 1:**
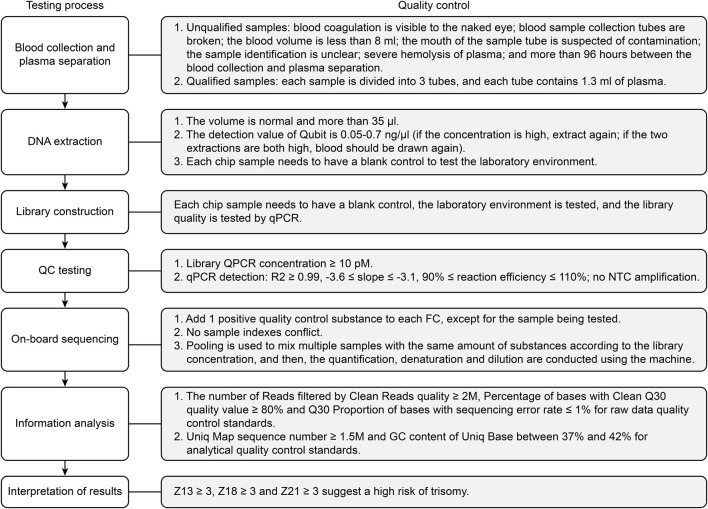
A flow chart of the NIPT testing process and quality control standards.

A total of 68,763 pregnant women who underwent NIPT with high risk cases, and prenatal diagnosis in the prenatal diagnosis agency of Beijing from January 2020 to December 2020 were enrolled in this study. The NIPT procedures, including the DNA extraction, library construction, whole genome sequencing, and data analysis, were carried out according to protocols published elsewhere (Liang et al., 2013). In brief, approximately 10 ml blood from each pregnant woman were collected into a purple-top tube containing EDTA. The maternal blood samples were subject to centrifugation at 1600 g for 10 min at 4°C, followed by 16000 g for 10 min at 4°C. The cffDNA was extracted from 1 ml plasma using a Circulating Nucleic Acid Kit from Berry Genomics. Subsequently, the extracted plasma DNA was used as the input DNA to prepare the library for sequencing. CffDNA was extracted using a DNA extraction kit (QIAGEN), and a DNA library was constructed using a library construction kit (Life Technologies). Quality control of the sequencing library was carried out using a QubitFluormeter quantifier. Sequencing detection was performed on an Illumina NextSeq CN500 or BerryGenomics NextSeq CN500, and bioinformatics analysis was performed using a proprietary algorithm. The binary hypothesis Z score of specific chromosomes in each sample was determined; the normal range for chromosomes was -3 < *z* < 3; a higher/lower score was classified as NIPT high-risk. A flowchart of the NIPT testing process and quality control standards is shown in Figure 1.

### Karyotype analysis and CNV

In total, 755 patients with high-risk NIPT results consented to undergo invasive prenatal diagnosis (741 cases underwent amniotic fluid puncture, and 14 cases under went cord blood puncture), which is the gold standard for diagnosing chromosome aneuploidies. Karyotype analysis at the 320-band level and/or copy number variation (CNV) on Illumina NextSeq CN500 andChAS2.0 was used to interpret CNVs, and data analysis was performed with reference to the online public database. A true-positive case was defined as a karyotype or CNV that was consistent with the NIPT result.

### Follow-up

Follow-up subjects were all people who underwent NIPT, including those with high-risk and low-risk NIPT results.

### Statistical analyses

All statistical analyses were performed using SPSS 18.0 software. A chi-squared test was used to assess the between-group differences. The 95% confidence interval (CI) was calculated to examine the positive rate (high-risk NIPT result/total number of NIPT tests), detection rate (sensitivity), positive predictive values (PPVs) and specificity. *p* < 0.05 was considered statistically significant.

## Results

### Basic characteristics of pregnant women who underwent NIPT

In this study, the age of pregnant women ranged from 18 to 45 years, with an average age of 32.0 ± 4.3 years. The gestational age at NIPT ranged from 12^+0^ to 26^+6^ weeks, with an average time of 16.3 ± 3.2 weeks. The gestational age of prenatal diagnosis ranged from 13^+0^ to 29^+6^ weeks, with an average time of 20.3 ± 4.4 weeks. Among 68,763 patients screened by NIPT, all subjects were grouped according to screening indications. The basic information and proportion of different indications are shown in Table 1.

**TABLE 1:** Basic information and proportion of different screening indications for NIPT.

Indications	Total number	Average age (year)	Average gestational age (week)	Population (%)
Advanced maternal age	22357	37.7±3.6	15.7±3.3	32.51
High risk of serological screening	1701	30.8±4.1	19.3±4.1	2.47
Critical risk of serological screening	8756	27.7±5.2	18.5±3.6	12.73
NT thickening	395	32.5±2.7	13.8±2.3	0.57
Ultrasound soft marker abnormality	182	31.8±3.2	23.8±1.9	0.26
Ultrasound structural abnormality	63	32.6±5.4	23.6±2.5	0.09
Twin/IVF-ET pregnancy	2666	28.4±3.1	15.5±2.3	3.88
Missed time for serological screening	274	32.9±2.9	23.4±2.8	0.40
Voluntary request	23346	29.3±5.2	15.6±3.1	33.95
Contraindications for interventional surgery	6	32.1±2.8	20.7±3.4	0.01
Others	9017	30.4±3.7	16.8±2.7	13.11
Total	68763	32.0±4.3	16.3±3.2	100.00

### Efficiency of NIPT for T21/T18/T13 and sex chromosome abnormalities

Among 68,763 pregnant women, 920 had high-risk results for T21/T18/T13/sex chromosome abnormalities (SCAs)/other chromosome abnormalities, including 164 with T21, 70 with T18, 38 with T13, 360 with SCAs (abnormal number of X or Y chromosomes) and 288 with other chromosome abnormalities. After informed consent was obtained, 755 patients accepted prenatal diagnosis by amniotic fluid cell analysis, and the rate of prenatal diagnosis was 82.07% (755/920). After follow-up, we identified 5 cases with false-negative results. Table 2 shows the prenatal diagnosis results. The detection rates of T21, T18 and T13 were 98.17% (107/109), 96.15% (25/26), and 100% (7/7), respectively. The PPVs of T21,T18,T13,SCAs and the others chromosome abnormalities were 65.24% (107/164), 35.71% (25/70), 18.42% (7/38),31.39% (113/360), and 18.75% (54/288), respectively, with significant differences (χ^2^ = 62.64, *p* < 0.001).

**TABLE 2:** NIPT positive results for T21/T18/T13/SCAs and other chromosome abnormalities.

NIPT result	High-risk result	True positive	False positive	False negative	Detection rate % (95%CI)	Specificity % (95%CI)	PPV % (95%CI)	*p*-value of PPV
T21	164	107	57	2	98.17 (93.53–99.78)	99.92 (99.89–99.94)	65.24 (57.43–72.50)	
T18	70	25	45	1	96.15 (80.36–99.90)	99.93 (99.91–99.95)	35.71 (24.61–48.07)	
T13	38	7	31	0	100 (59.04–100)	99.95 (99.94–99.97)	18.42 (7.74–34.33)	< 0.001
T21/18/13	272	139	133	3	97.89 (93.95–99.56)	99.81 (99.77–99.84)	51.10 (44.99–57.19)	
SCAs	360	113	247	-	-	-	31.39 (26.63–36.46)	
Other chromosomal abnormalities	288	54	184	-	-	-	18.75 (14.41–23.75)	

### False-negative NIPT cases

We identified 5 cases with false-negative NIPT results. Case 1 with indications for NIPT voluntarily requested the analysis. In follow-up ultrasound screening, it was found that the width of the foetal lateral ventricle was more than 10 mm; therefore, the prenatal diagnosis was T21. Case 2 involved advanced maternal age (≥35 years) and nuchal translucency (NT) thickening. In follow-up ultrasound screening, a choroid plexus cyst was detected, and the karyotype analysis was T21. T18 was observed in case 3. The woman voluntarily requested NIPT; in follow-up ultrasound screening, it was revealed that she had a single umbilical artery and interventricular septal defect but refused a prenatal diagnosis. Therefore, a chromosome examination was performed after delivery, and the result was T18. The other 2 cases involved SCAs; both patients were of advanced maternal age, and the karyotype analysis results were 47,XXY (Table 3).

**TABLE 3:** Details of the false-negative NIPT cases.

Case number	Maternal age (year)	Ultrasound screening abnormalities	Diagnosis methods	cffDNA content (%)	Prenatal diagnosis results
1	28	Foetal lateral ventricle was more than 10 mm and increased with gestational age	Cord blood puncture	9.63	T21
2	37	NT thickening and choroid plexus cyst	Amniotic fluid puncture	7.38	T21
3	33	Ultrasound structural abnormality	Newborn chromosome	6.63	T18
4	38	Ultrasound soft marker abnormality	Amniotic fluid puncture	7.64	47, XXY
5	35	Ultrasound soft marker abnormality	Amniotic fluid puncture	8.95	47, XXY

### Distribution of indications

Among the 68,763 prenatal women who received NIPT, the positive rate, detection rate and PPV differed among different NIPT indications (Table 4 and Table 5). Regarding the positive rates, the top three indications for NIPT among the cases ofT21 were NT thickening (2.03%,8/395), ultrasound structural abnormalities (1.59%,1/63) and ultrasound soft marker abnormalities (1.10%,2/182). Additionally, the detection indication with the highest rate among T18, T13, and SCA cases was ultrasound structural abnormalities. Furthermore, the NIPT detection indications with the highest PPVs (100%) were ultrasound structural abnormalities and ultrasound soft marker abnormalities for T21 and SCAs, although the PPVs (100%) of T18 and T13 with ultrasound structural abnormalities or NT thickening were the highest. Additionally, the PPVs of all clinical indications forT21 (χ^2^ = 22.40, *p* = 0.002), T13 (χ^2^ = 11.06, *p* = 0.04) and SACs (χ^2^ = 17.55, *p* = 0.02), but notT18 (χ^2^ = 10.41, *p* = 0.18), were statistically significant. The positive rate among pregnant women with a high risk of serological screening (0.76%, 13/1701) was higher than that for the other detection indications (except ultrasound abnormalities), and the detection rate was 100% for T21,T18,and SCAs. However, those who voluntarily requested NIPT had the lowest detection rate. Last, the NIPT detection rate and PPV of T21 were higher than those of T18, T13 or SCAs.

**TABLE 4:** Aneuploidy positive rates according to NIPT screening indications.

Indications	Total number	High-risk NIPT cases				True positive cases				False-negative cases				NIPT Positive rate% (95%CI)			
		T21	T18	T13	SCAs	T21	T18	T13	SCAs	T21	T18	T13	SCAs	T21	T18	T13	SCAs
Advanced maternal age	22357	74	32	9	133	57	14	3	47	1	0	0	2	0.33 (0.26-0.42)	0.14 (0.10-0.20)	0.04 (0.02-0.08)	0.59 (0.50-0.70)
High risk of serological screening	1701	13	6	2	9	6	2	0	3	0	0	0	0	0.76 (0.41-1.30)	0.35 (0.13-0.77)	0.12 (0.01-0.42)	0.53 (0.24-1.00)
Critical risk of serological screening	8756	12	2	8	40	6	0	1	7	0	0	0	0	0.14 (0.07-0.24)	0.02 (0-0.08)	0.09 (0.04-0.18)	0.46 (0.33-0.62)
NT thickening	395	8	8	4	2	7	5	2	2	0	0	0	0	2.03 (0.88-3.95)	2.03 (0.88-3.95)	1.01 (0.28-2.57)	0.51 (0.06-1.81)
Ultrasound soft marker abnormality	182	2	2	0	1	2	0	0	0	0	0	0	0	1.1 (0.13-3.91)	1.1 (0.13-3.91)	0 (0-2.01)	0.55 (0.01-3.02)
Ultrasound structural abnormality	63	1	1	1	1	1	1	1	1	0	0	0	0	1.59 (0.04-8.53)	1.59 (0.04-8.53)	1.59 (0.04-8.53)	1.59 (0.04-8.53)
Twin/IVF-ET pregnancy	2666	6	3	0	16	3	1	0	6	0	0	0	0	0.23 (0.08-0.49)	0.11 (0.02-0.33)	0 (0-0.14)	0.60 (0.34-0.97)
Missed time for serological screening	274	1	0	0	2	0	0	0	2	0	0	0	0	0.36 (0.01-2.02)	0 (0-0.14)	0 (0-0.14)	0.73 (0.09-2.61)
Voluntary request	23346	41	10	13	120	19	2	0	31	1	1	0	0	0.18 (0.13-0.24)	0.04 (0.02-0.08)	0.06 (0.03-0.10)	0.51 (0.13-0.61)
Contraindications for interventional surgery	6	0	0	0	0	0	0	0	0	0	0	0	0	0 (0-0.46)	0 (0-0.46)	0 (0-0.46)	0 (0-0.46)
Others	9017	6	6	1	36	6	0	0	14	0	0	0	0	0.07 (0.02-1.44)	0.07 (0.02-1.44)	0.01 (0-0.06)	0.40 (0.28-0.55)
Total	68763	164	70	38	360	107	25	7	113	2	1	0	2	0.24 (0.20-0.28)	0.10 (0.08-0.13)	0.06 (0.04-0.08)	0.52 (0.47-0.58)

**TABLE 5:** Aneuploidy detection rates according to NIPT screening indications.

Indications	NIPT detection rate% (95%CI)				NIPT PPV% (95%CI)			
	T21	T18	T13	SCAs	T21	T18	T13	SCAs
Advanced maternal age	98.28 (90.76–99.96)	100 (76.84–100)	100 (29.24–100)	95.92 (86.02–99.50)	77.03 (65.79–86.01)	43.75 (26.36–62.34)	33.33 (7.49–70.07)	35.34 (27.25–44.09)
High risk of serological screening	100 (54.07–100)	100 (15.81–100)	/	100 (29.24–100)	46.15 (19.22–74.87)	33.33 (4.33–77.72)	0 (0–84.19)	33.33 (7.49–70.07)
Critical risk of serological screening	100 (54.07–100)	/	100 (2.50–100)	100 (59.04–100)	50 (21.09–78.91)	0 (0–84.19)	12.50 (0.32–52.65)	17.50 (7.34–32.78)
NT thickening	100 (59.04–100)	100 (47.82–100)	100 (15.81–100)	100 (15.81–100)	87.5 (47.35–99.68)	62.5 (24.49–91.48)	50.00 (6.76–93.24)	100 (15.81–100)
Ultrasound soft marker abnormality	100 (15.81–100)	/	/	/	100 (15.81–100)	0 (0–84.19)	/	090–97.50)
Ultrasound structural abnormality	100 (2.50–100)	100 (2.50–100)	100 (2.50–100)	100 (2.50–100)	100 (2.5–100)	100 (2.5–100)	100 (2.5–100)	100 (2.5–100)
Twin/IVF-ET pregnancy	100 (29.24–100)	100 (2.50–100)	/	100 (54.07–100)	50 (11.81–88.19)	33.33 (0.84–90.57)	/	37.50 (15.20–64.57)
Missed time for serological screening	/	/	/	100 (15.81–100)	0 (0–97.50)	/	/	100 (15.81–100)
Voluntary request	95.00 (75.13–99.87)	66.67 (9.43–99.16)	/	100 (88.78–100)	46.34 (30.66–62.58)	20.00 (2.52–55.61)	0 (0–24.71)	25.83 (18.28–34.62)
Contraindications for interventional surgery	/	/	/	/	/	/	/	/
Others	100 (54.07–100)	/	/	100 (76.84–100)	100 (54.07–100)	0 (0–45.93)	0 (0–97.50)	38.89 (23.14–56.54)
Total	98.17 (93.53–99.78)	96.15 (80.36–99.90)	100 (59.04–100)	98.26 (93.86–99.79)	65.24 (57.43–72.50)	35.71 (24.61–48.07)	18.42 (7.74–34.33)	31.39 (26.63–36.46)

### Comparison of low-risk and high-risk pregnant women

Among 68,763 cases, 23,346 (33.95%, 23,346/68,763) were at low risk (voluntary request), and 45,417 (66.05%, 4517/68,763) were at high risk. The positive rate (0.46% vs. 0.27%, *p* < 0.001), PPV (56.73% vs 32.81%, *p* = 0.001) and detection rate (99.16% vs. 91.30%, *p* = 0.02) of T21, 18 and 13 in high-risk pregnant women were higher than those in low-risk pregnant women (all *p* < 0.05), but there was no significant difference in specificity between them (χ2 = 0.16, *p* = 0.71). For SCAs, there was no statistically significant difference in screening efficiency between the two groups. The screening efficiency of T21, T18, T13 and SCAs in both groups are shown in Table 6 and Table 7.

**TABLE 6:** Comparison of the screening efficiency of NIPT between low-risk and high-risk pregnancy women.

Indications	Total number	High-risk NIPT cases					True positive cases					False-negative cases					NIPT Positive rate% (95%CI)				
		T21	T18	T13	SCAs	T21/18/13	T21	T18	T13	SCAs	T21/18/13	T21	T18	T13	SCAs	T21/18/13	T21	T18	T13	SCAs	T21/18/13
High risk	45417	123	60	25	240	208	88	23	7	82	118	1	0	0	2	1	0.27 (0.23–0.32)	0.13 (0.10–0.17)	0.06 (0.04–0.08)	0.53 (0.46–0.60)	0.46 (0.40–0.52)
Voluntary request	23346	41	10	13	120	64	19	2	0	31	21	1	1	0	0	2	0.18 (0.13–0.23)	0.04 (0.02–0.07)	0.06 (0.03–0.10)	0.51 (0.43–0.61)	0.27 (0.21–0.35)
χ (Zhang et al., 2015)																	5.87	12.09	0.001	0.06	13.23
*p* value																	0.02	0.001	1	0.82	<0.001

**TABLE 7:** Comparison of the screening efficiency of NIPT between low-risk and high-risk pregnancy women.

Indications	NIPT detection rate% (95%CI)					NIPT PPV% (95%CI)					NIPT specificity % (95%CI)			
	T21	T18	T13	SCAs	T21/18/13	T21	T18	T13	SCAs	T21/18/13	T21	T18	T13	T21/18/13
High risk	98.88 (93.90–99.97)	100 (85.18–100)	100 (59.04–100)	97.62 (91.66–99.71)	99.16 (95.41–99.98)	71.54 (62.71–79.31)	38.33 (26.07–51.79)	28.00 (12.07–49.39)	34.17 (28.19–40.54)	56.73 (49.70–63.56)	99.92 (99.89–99.94)	99.92 (99.89–99.94)	99.96 (99.94–99.98)	99.80 (99.76–99.84)
Voluntary request	95.00 (75.13–99.87)	66.67 (9.43–99.16)	100 (2.50–100)	100 (88.78–100)	91.30 (71.96–98.93)	46.34 (30.66–62.58)	20.00 (2.52–55.61)	0.00 (0–24.71)	25.83 (18.28–34.62)	32.81 (21.59–45.69)	99.91 (99.86–99.94)	99.97 (99.93–99.99)	99.94 (99.90–99.97)	99.82 (99.75–99.87)
χ (Zhang et al., 2015)	1.36	8	0.001	0.75	5.75	16.95	1.3	4.46	2.60	11.21	0.54	5.25	0.88	0.16
*p* value	0.24	0	1	0.39	0.02	<0.001	0.3	0.04	0.10	0.001	0.46	0.22	0.35	0.71

## Discussion

Diseases of chromosomal aneuploidy, including Down syndrome (trisomy 21 or T21), Edward syndrome (trisomy 18 or T18), Patau syndrome (trisomy 13 or T13) and sex chromosome abnormalities (SCAs), are characterized by more than 23 pairs of chromosomes. SCAs include Turner syndrome (45,X), Klinefelter syndrome (47,XXY), Triple X syndrome (47,XXX) and 47,XYY syndrome (47,XYY). These chromosomal anomalies contribute to morbidity and death in both childhood and adulthood (Badeau et al., 2017).

According to our clinical data from 68,763 cases, the detection rate/specificity was 98.17%/99.92% for T21, 96.15%/99.93% for T18 and 100%/99.95% for T13. These findings are consistent with those of other studies (Zheng et al., 2019), even though the PPV for T21 was lower (Junhui et al., 2021; Tekesin, 2021). Compared with serological screening, the detection rate and specificity of NIPT in screening for T21 were greater (Shaw et al., 2013). For T18, the specificity was 99.93%, and the PPV was 35.71%; for T13, the PPV was 18.42%, which was similar to that in other studies (Xue et al., 2019). Compared to a previous study (Lund et al., 2021), the results for T13 were not correlated which may be related to the small number of NIPT-positive cases. In the future, more cases should be used to evaluate the effectiveness of NIPT for detecting T13.

There were 360 cases with NIPT-positive SCA results, and 113 cases were true-positive results. The overall positive rate and PPV for SCA detection by NIPT were 0.52% and 31.39%, respectively. The positive rate was higher and the PPV lower than in previous research (Cheung et al., 2015; Suo et al., 2018; Yin et al., 2020). We observed that pregnant women whose NIPT results were associated with SCAs were inclined to reject prenatal diagnosis and choose direct delivery. Possible reasons are as follows. First, NIPT has a high false-positive rate for SCAs. Second, families are more likely to accept children with SCAs. According to our results, the PPV for SCAs was low, which may be related to the low prenatal diagnosis rate; the standard methods of follow-up is not accurate with false-negative results for SCA disease because SCA babies are usually mildly symptomatic or asymptomatic during the neonatal period, without any physical or intellectual disabilities. Therefore, we did not analyse the sensitivity and specificity of SCAs in this report. In the interest of the health of the baby, women carrying a foetus with a positive result for SCA are advised to undergo a prenatal diagnosis to determine the foetal karyotype. Pregnant women at high risk for other chromosomal abnormalities are more likely to refuse prenatal diagnosis, which may be a factor in the low PPV (Ying et al., 2020); relatedly, in cases of abortion before diagnosis, a chromosome diagnosis of the aborted tissue was not performed, which may be due to a lack of understanding of NIPT among pregnant women. In our report, among the 288 patients with high risk for other chromosomal abnormalities, 54 patients had true positives and terminated their pregnancy, 184 patients had false positives and live births, and eight patients terminated their pregnancy, of which 3 patients were complicated with abnormal ultrasound structure. Three patients had embryos that had stopped developing. The other 39 patients were lost to follow-up. In addition, we do not know the number of false negatives. Therefore, the reporting mechanism of birth defects with other chromosomal abnormalities is not perfect in Beijing, and errors or omissions may be reported, so we did not analyse and discuss them in this study.

In our study, there were five cases of false-negative NIPT, including 2 with T21 and 1 with T18. NIPT has high sensitivity and specificity for T21 and T18, but they also have a risk of missed diagnosis. Among the causes of false-negative results of NIPT, the common reasons are low cffDNA content, placental mosaicism and inconsistency of foetal-placental DNA Lin et al. (Lin et al., 2021) reported that false negatives occur easily when the foetal DNA content is less than 2%. In our study, we included strict quality control and excluded factors that may affect the test results, such as gestational age, weight, cffDNA, etc. We excluded low cffDNA content because when cffDNA is lower than 4%, the detection instrument prompts detection failure and cannot issue an experimental report. Through the detection of foetal and placental DNA, we found one case of low-proportion mosaicism of foetal T21 because when the mosaicism level is low, abnormal DNA may not be detected in the peripheral blood of pregnant women (Canick et al., 2013). The other two cases were placental mosaicism. Previous studies have reported false-negative NIPT caused by placental mosaicism (Pan et al., 2014). Hence, it is very important to conduct adequate clinical consultation before testing, and it is necessary to reduce the false-positive rate and encourage pregnant women with positive NIPT results to undergo further prenatal diagnosis.

In our study, we also analysed the distribution of the indications for NIPT and the positive rate and detection rate of each indication, this is the highlight of this study. We refined the screening indications for NIPT and analyzed its detection efficacy. The results showed that the highest positive rate and PPV of NIPT detection indications were both related to ultrasound abnormalities, especially ultrasound structural abnormalities and NT thickening; those results are similar to the previously reported data published by Wang et al. (2021). These results showed that pregnant women with abnormal ultrasound results are more likely to be carrying a foetus with chromosomal abnormalities, and they should be diagnosed. If those women are offered NIPT first, there would be a significant diagnostic delay because all abnormal NIPT results need to be confirmed by diagnostic testing (Petersen et al., 2020). Therefore, the prenatal diagnosis is more accurate and preferable for pregnant women with abnormal ultrasound structures. Based on our results, the detection rate of NIPT in pregnant women at an advanced age was more than 98%, and the PPV was 77.03% for T21, which was higher than that in low-risk cases (voluntary request group), with a detection rate of 95% and a PPV of 46.34%.These results demonstrate that NIPT is more suitable for high-risk populations. However, the current research focused mostly on high-risk pregnant women, such as those with advanced age, but this has not been fully studied in low-risk populations. Therefore, we analysed the low-risk cases and compared them with the high-risk cases (except voluntary requests). We found that except for the specificity, the positive rate (0.46% vs. 0.27%), detection rate (99.16% vs.91.30%), and PPV (56.73% vs.32.81%) of T21/18/13 in the high-risk group were all higher than those in the low-risk group. According to our findings, NIPT screening in a low-risk population will reduce the sensitivity and PPV of NIPT, which is basically consistent with the results of previous studies (Committee Opinion No. 640, 2015; Wang et al., 2019). If low-risk pregnant women are advised to undergo NIPT, the detection rate and PPV should be informed in detail before testing, and high-risk cases should undergo prenatal diagnosis. If guidelines expand NIPT recommendations to include low-risk patients, the economic benefits should be further evaluated.

## Conclusion

Although the high sensitivity and specificity of NIPT have great advantages, the disadvantage of NIPT is obvious, namely, false positives and false negatives. Therefore, doctors should clearly inform patients about the limitations of NIPT, including the detection rate, positive rate, and PPV, among others, before testing. In general, we should advocate more caution for people who need a prenatal diagnosis to undergo NIPT, especially in pregnant women at an advanced age or with ultrasound structural abnormalities. NIPT is a potential method for foetal SCA detection, in addition to common aneuploidy screening. However, application of this technique needs to be further investigated. Moreover, we should deepen the analysis of clinical data to explore the clinical indications of NIPT and combine the economic benefits to determine whether NIPT is more advantageous for low-risk populations.

## Data Availability

The original contributions presented in the study are included in the article/supplementary material, further inquiries can be directed to the corresponding author.
